# Supplementary material for: Assembly-free rapid differential gene expression analysis in non-model organisms using DNA-protein alignment
Source: BMC Genomics. 2022 Feb 4;23:97. doi: 10.1186/s12864-021-08278-7 (PMC8815227; doi:10.1186/s12864-021-08278-7)
Supplement: Supplementary file 1 — Additional file 1 Supplementary material. [file 12864_2021_8278_MOESM1_ESM.pdf]

Rapid differential gene expression analysis in non-model organisms using DNA-protein alignment: Supplementary Material

1 Computational pipelines

Shown below are the computational pipelines used in our work. All the pipelines described below are available at the following repository: `https://bitbucket.org/project_samar/benchmarking`.

Our approach

We used LAST (version 1060). An index of the reference proteome was created:

```
lastdb -p index reference.fa
```

A sample of 100,000 reads were in-silico translated in all 6 frames, and the translated sequences were used to learn the scoring scheme:

```
last-train --revsym --matsym --gapsym --sample-number=600000 -S0 index \
    translated_sequence.fa > scoring_scheme
```

Fragment size mean and standard deviation was estimated by aligning a sample of the reads.

```
head -n 200000 reads1_1.fa > sample1_1.fa
head -n 200000 reads1_2.fa > sample1_2.fa
fasta-interleave sample1.fa sample2.fa | lastal -i1 -p scoring_scheme -F 20 \
    db |  last-pair-probs -e
```

The reads were aligned to the reference using the paired-end information:

```
fasta-interleave reads1.fa reads2.fa | lastal -i1 -p scoring_scheme -F15 index | \
last-pair-probs -f fragment_size_mean -s fragment_size_std -m 0.95 -d 0.1 | \
maf-convert tab > alignments
```

Count data was computed :

```
python count.py alignments counts --frag_len_mean fragmen_size_mean \
    --frag_len_std --reference reference.fa
```

Our count file format mimics that of Salmon, so that it can be read in by tximport, before calling DESeq2 for differential expression analysis.

```
txi <- tximport(files_counts, type = "salmon", txIn = TRUE, txOut = TRUE)
ourMethod_dds <- DESeqDataSetFromTximport(txi, colData = colData, design = ~ condition)
ourMethod_dds <- DESeq(ourMethod_dds)
```

Assembly-based approach

De-novo transcriptome assembly was computed from all the reads in the dataset using Trinity (version 2.8.5):

```
Trinity --seqType fa --max_memory 90G --left reads1_1.fa,..,reads6_1.fa \
        --right reads1_2,..,reads1_6.fa --CPU 20
```

The reads were aligned to the assembled transcripts using Bowtie2 (version 2.4.1):

```
bowtie2-build Trinity.fasta index
bowtie2 -p 20 -x index -1 reads1_1.fa -2 reads1_2.fa --no-mixed \
     --no-discordant --gbar 100 --end-to-end -k 200 | \
     samtools view -bS - > alns.bam
```

Counting was done using RSEM:

```
rsem-prepare-reference Trinity.fasta rsem_index
rsem-calculate-expression -q --no-bam-output --alignments --paired-end alns.bam rsem_index [illegible]
```

Transcript-level counts were aggregated at the gene level using tximport, based on the gene-transcript map constructed by Trinity

```
contig2gene <- read.delim("Trinity.fasta.gene_trans_map", header=FALSE)[,c(2,1)]
txi <- tximport(files_counts, type = "rsem", txIn = TRUE, txOut = FALSE, tx2gene = contig2g[illegible]

assembly_dds <- DESeqDataSetFromTximport(txi, colData = colData, design = ~ condition)
assembly_dds <- DESeq(assembly_dds)
```

Baseline

The Bowtie2-RSEM-DESeq2 pipeline was run as above but with the *D. melanogaster* transcriptome as the reference.

2 Isoform-level quantification

To supplement our finding on gene-level counting by using a reference with 1 representative protein sequence per gene, we used the Uniprot fruit fly proteome containing sequences of all isoforms as the reference. After removing duplicates and ones with no corresponding Flybase ID, there were 19,828 sequences in the proteome. The non-redundant protein was used as reference to align the simulated reads. Prior to differential analysis using DESeq2, we aggregated the isoform-level counts to gene level using tximport based on gene-to-protein mapping obtained from Ensembl. We tried both the simple sum mode and scaled TPM mode of tximport (although our method does not techically compute TPM, since we use the length of the CDS region). Figure 1 shows the performance using the same evaluation metric as in the main text. We see no major differences.

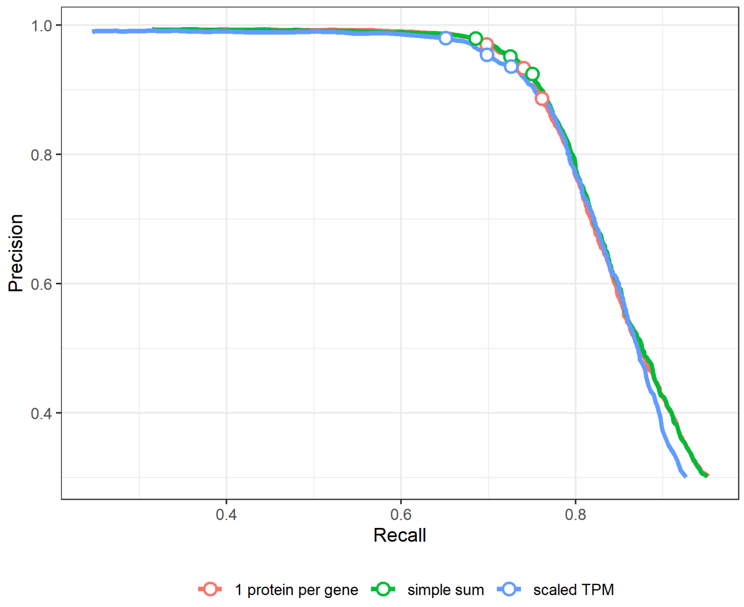

Figure 1: Isoform-level quantification followed by gene-level inference of differential expression.
